# Role of Adjuvant Chemotherapy in Resected Small Bowel Adenocarcinoma: An Exploratory Real-World Analysis of Survival Outcomes and Prognostic Factors

**DOI:** 10.3390/jcm14217513

**Published:** 2025-10-23

**Authors:** Jirapat Wonglhow, Patrapim Sunpaweravong, Chirawadee Sathitruangsak, Arunee Dechaphunkul, Panu Wetwittayakhlang

**Affiliations:** 1 Division of Medical Oncology, Department of Internal Medicine, Faculty of Medicine, Prince of Songkla University, Songkhla 90110, Thailand; jirapat.jw@gmail.com (J.W.); spatrapi@medicine.psu.ac.th (P.S.); sjirawadee@gmail.com (C.S.); dr.arunee@gmail.com (A.D.); 2 Gastroenterology and Hepatology Unit, Division of Internal Medicine, Faculty of Medicine, Prince of Songkla University, Songkhla 90110, Thailand

**Keywords:** small bowel cancer, adenocarcinoma, early stage, adjuvant chemotherapy, prognosis, survival

## Abstract

**Background**: Small bowel adenocarcinoma (SBA) is a rare malignancy, and the role of adjuvant chemotherapy following curative resection remains unclear owing to limited supporting evidence. In this study, we aimed to evaluate the real-world effectiveness of adjuvant chemotherapy in patients with resected SBA. **Methods**: We retrospectively reviewed data from patients with localized SBA who underwent curative resection at a single tertiary referral center in Southern Thailand between 2005 and 2024. **Results**: Of 128 patients diagnosed with SBA, 52 (40.6%) had localized disease and underwent curative resection. Among them, 29 patients (55.8%) received adjuvant chemotherapy and 23 (44.2%) were managed with observation alone. The median disease-free survival (DFS) was 18.1 and 16.2 months in the adjuvant chemotherapy and observation groups, respectively (*p* = 0.642). The median overall survival (OS) was 42.8 vs. 26.7 months, respectively (*p* = 0.179). Subgroup analyses revealed trends favoring adjuvant chemotherapy in patients with pathological T4 disease, nodal involvement, younger age, and non-underweight body mass indices. Positive surgical margins were associated with inferior DFS, and T4 stage was associated with worse OS. Disease recurrence occurred in 59% of patients, predominantly as distant metastasis. **Conclusions**: Adjuvant chemotherapy showed a trend toward improved survival, particularly in patients with high-risk features; however, these findings should be interpreted with caution given the limited sample size and retrospective design. These results highlight the importance of individualized treatment decisions and underscore the need for larger multi-institutional studies to clarify the role of adjuvant chemotherapy and identify prognostic biomarkers for this rare malignancy.

## 1. Introduction

Small-bowel cancer is a rare malignancy that accounts for less than 5% of all gastrointestinal cancers, despite the small intestine being the longest segment of the digestive tract [1]. Small bowel adenocarcinoma (SBA) accounts for approximately 30–45% of small bowel cancers [2] with the duodenum being the most frequently affected site, followed by the jejunum and ileum [3]. Owing to its vague and nonspecific symptoms, SBA is often diagnosed at an advanced stage. Nonetheless, a subset of patients present with localized diseases amenable to curative surgical resection [2].

Currently, complete surgical resection with regional lymph node dissection is the only potentially curative treatment for localized SBA. However, recurrence rates remain high, particularly in node-positive patients [4]. Local recurrence, typically in the surgical bed and regional lymph nodes, occurs in approximately 30% of cases, whereas distant metastases are reported in 80–100% of relapses [5]. These recurrence patterns suggest that systemic adjuvant chemotherapy may reduce relapse rates and improve survival outcomes.

Due to the rarity of SBA, there is a lack of prospective randomized controlled trials (RCTs) that specifically evaluate the benefits of adjuvant chemotherapy. Consequently, current treatment practices are largely extrapolated from colorectal cancer management guidelines [6]. Some retrospective studies have suggested that adjuvant chemotherapy has a survival benefit, especially among patients with stage III disease or high-risk features, such as T4 tumors [7,8,9,10]. However, other studies have failed to demonstrate significant benefits [11,12,13,14,15], leaving the role of adjuvant chemotherapy unclear.

Given the conflicting retrospective evidence and absence of RCTs, the role of real-world data remain critical in informing clinical decision-making regarding the use of adjuvant chemotherapy in localized SBA treatment post-resection. Therefore, we conducted a retrospective cohort study, aiming to assess the disease-free survival (DFS) and overall survival (OS) in patients with resected SBA who received adjuvant chemotherapy and those managed with observation alone and to explore potential prognostic factors.

## 2. Materials and Methods

### 2.1. Study Participants and Procedures

In this retrospective study, we reviewed the medical records of patients diagnosed with SBA who underwent treatment at the Songklanagarind Hospital, Prince of Songkla University, between January 2005 and December 2024. Eligible patients were identified based on the following criteria: (1) histopathological confirmation of adenocarcinoma originating from the small intestine (duodenum, jejunum, or ileum); (2) receipt of curative-intent surgical resection; (3) pathological stage I–III disease according to the American Joint Committee on Cancer staging system, 8th edition; and (4) age of 18 years or more at diagnosis. Patients were excluded if they had (1) metastatic disease at presentation; (2) unresectable locally advanced tumors; or (3) received preoperative chemotherapy or radiotherapy.

Demographic and clinical data of the patients were systematically collected from the electronic hospital records. The key variables included age at diagnosis, sex, body mass index (BMI), Eastern Cooperative Oncology Group performance status, anatomical site of the primary tumor, pathological TNM classification, histological grade, serum tumor markers, treatment history, and follow-up information.

The study protocol was reviewed and approved by the Ethics Committee of the Research Center of the Faculty of Medicine, Prince of Songkla University (REC.68204141). The requirement for written informed consent was waived owing to the retrospective nature of the study. All data were anonymized to ensure patient confidentiality.

### 2.2. Outcome Measures

The primary aim of this study was to evaluate the DFS in patients with resected SBA who received adjuvant chemotherapy and those managed with observation alone. The secondary objectives were to evaluate the OS in the two groups and explore potential prognostic factors associated with DFS and OS. DFS was defined as the interval from the date of surgical resection to the first occurrence of radiologically confirmed disease recurrence or death from any cause, whichever occurred earlier. OS was defined as the time from surgical resection to death from any cause. Patients were monitored through routine clinical follow-up conducted every 3 months, with surveillance imaging, including chest and abdominal computed tomography, performed at 6-month intervals in the first 2 years and annually thereafter.

### 2.3. Statistical Analysis

Descriptive statistics were used to summarize the baseline characteristics. Continuous variables are reported as median with interquartile range or mean with standard deviation, depending on the data distribution. Categorical variables were presented as counts and percentages. Survival outcomes were estimated using the Kaplan–Meier method, and comparisons between groups were performed using the log-rank test. Multivariate analyses were conducted using Cox proportional hazards regression models to account for potential confounding factors influencing survival outcomes. Univariate and multivariate Cox regression analyses were used to identify the independent prognostic factors. Relevant potential variables were selected from the database considering both clinical and statistical aspects from the literature review. Variables with a *p*-value < 0.20 in univariate analysis were subsequently included in the multivariate model. The proportional hazards assumption for each covariate was tested using Schoenfeld residuals. All statistical analyses were performed using the R software version 4.3.1 (R Foundation for Statistical Computing, Vienna, Austria). Two-sided *p*-values were reported, with statistical significance threshold set at *p*-value < 0.05.

## 3. Results

### 3.1. Baseline Characteristics

A total of 128 patients with SBA were identified, of whom 52 (40.6%) were diagnosed with localized disease and underwent curative surgical resection. Among these 52 patients, 29 (55.8%) received adjuvant chemotherapy, whereas 23 (44.2%) were managed with observation alone. The cohort had an even sex distribution and a mean age of 60.4 years. The duodenum was the most common primary tumor site (75.0%), followed by the jejunum (17.3%) and ileum (7.7%). The baseline characteristics were generally well balanced between the two groups, except for pathological nodal and surgical margin status (Table 1). Patients in the adjuvant chemotherapy group had a higher frequency of lymph node positivity and positive resection margins than did those in the observation group.

### 3.2. Treatment Information

Among the patients who received adjuvant chemotherapy, the majority (89.7%) were treated with chemotherapy alone, whereas three patients (10.3%) with positive surgical margins underwent concurrent chemoradiotherapy. All the patients were treated with fluoropyrimidine-based regimens. Approximately 41.3% of patients received an oxaliplatin-based doublet regimen followed by single-agent chemotherapy, as summarized in Table 2. Overall, 69% of the patients completed the planned 6-month course of adjuvant therapy, whereas the remainder discontinued treatment owing to disease recurrence, their preference, or intolerance to adverse effects.

### 3.3. DFS

**Figure 1:**
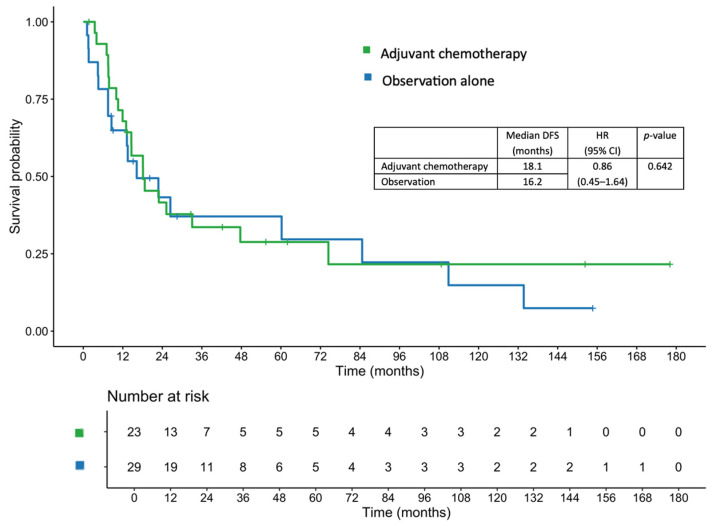
Disease-free survival in patients who received adjuvant chemotherapy and those managed with observation alone.

The median patient follow-up time was 27.23 (range, 1.74–178.09) months. In the adjuvant chemotherapy and observation cohorts, the median follow-up times were 32.69 (range, 1.74–178.09) and 16.23 (range, 2.37–154.67) months, respectively. The median DFS in the entire cohort was 18.1 months. Patients in the adjuvant chemotherapy group had a median DFS of 18.1 months, compared with 16.2 months among those in the observation group (hazard ratio [HR], 0.86; 95% confidence interval [CI], 0.45–1.64; *p* = 0.642; Figure 1).

After adjustment for covariates including age, sex, BMI, tumor differentiation, pathological T and N stage, lymphovascular invasion (LVI), and margin status using a multivariable Cox proportional hazards regression model, the adjusted HR was 0.81 (95% CI, 0.32–2.04; *p* = 0.651). The 1-, 2-, and 3-year DFS rates in the adjuvant chemotherapy group were 67.9%, 41.6%, and 33.6%, respectively, compared with 64.9%, 43.3%, and 37.1%, respectively, in the observation group.

**Figure 2:**
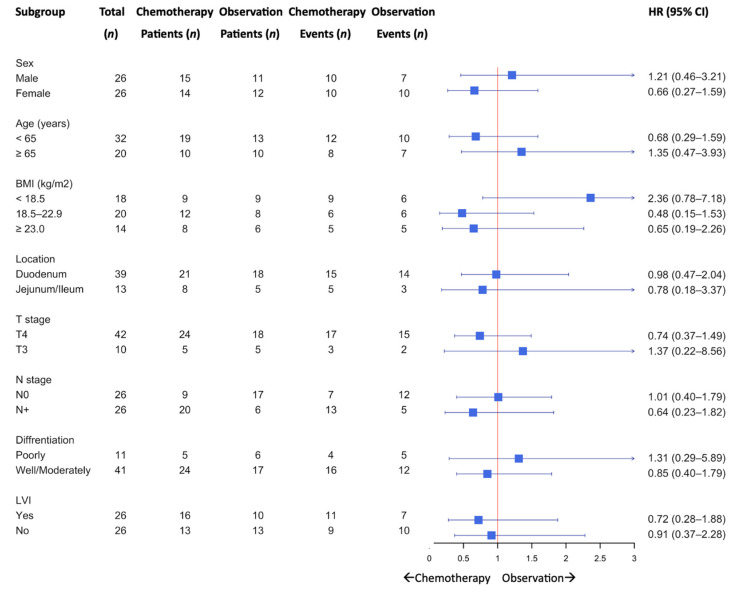
Subgroup analysis for disease-free survival.

A subgroup analysis of DFS is shown in Figure 2. Although no statistically significant differences were observed across the subgroups, a trend favoring adjuvant chemotherapy was observed among patients aged < 65 years, those with non-underweight BMI, T4 tumors, and nodal involvement (N+).

Approximately 60% of the patients experienced disease recurrence. The most common pattern of recurrence was distant metastasis (Table 3), with the peritoneum being the most frequent site, followed by the lymph nodes and liver. Local recurrence in the surgical bed was observed in approximately 10% of patients. There was no statistically significant difference in the recurrence patterns between the two groups. Subsequent palliative systemic chemotherapy was administered to 78.9% of the patients in the adjuvant chemotherapy group and 50.0% of those in the observation group. Details of palliative treatments are provided in Table S1. Notably, one patient in the adjuvant chemotherapy group underwent liver metastasectomy after receiving palliative chemotherapy and subsequently exhibited no evidence of disease, remaining under surveillance with no further recurrence.

### 3.4. OS

**Figure 3:**
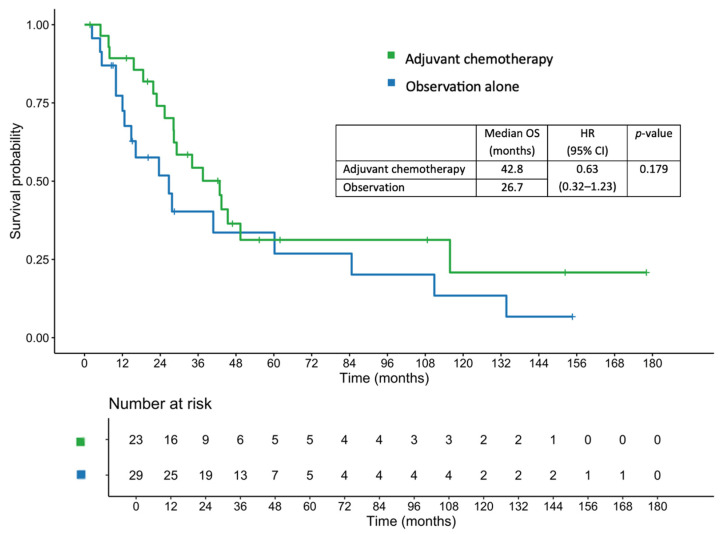
Overall survival in patients who received adjuvant chemotherapy and those managed with observation alone.

The median OS in the entire cohort was 34.1 months. The median OS was 42.8 months in the adjuvant chemotherapy group, compared with 26.7 months in the observation group (HR, 0.63; 95% CI, 0.32–1.23; *p* = 0.179; Figure 3). After adjustment using a multivariate Cox proportional hazards regression model, the adjusted HR was 0.61 (95% CI, 0.22–1.74; *p* = 0.357). The 1-, 2-, and 3-year OS rates in the adjuvant chemotherapy group were 89.3%, 74.1%, and 54.3%, respectively, compared with 77.3%, 51.8%, and 40.3%, respectively, in the observation group.

In the lymph node-positive subgroup, patients receiving adjuvant chemotherapy had a median OS of 37.5 months, compared with 23.6 months in those who were under observation alone (HR, 0.53; 95% CI, 0.18–1.52; *p* = 0.238). Similarly, among patients with pathological T4 disease, the median OS was 37.5 months in the adjuvant chemotherapy group versus 23.6 months in the observation group (HR, 0.58; 95% CI, 0.28–1.18; *p* = 0.130).

### 3.5. Prognostic Factors

Multivariate Cox proportional hazards regression analysis identified positive surgical margins as significant predictors of shorter DFS, whereas pathological T4 stage was significantly associated with worse OS (Table 4). The proportional hazards assumption was verified using Schoenfeld residuals, which showed no violation for any covariate (global test *p* = 0.72).

Elevated levels of tumor markers, including carcinoembryonic antigen (CEA) and carbohydrate antigen 19-9 (CA19-9), were associated with poor DFS and OS. However, owing to limited data availability, tumor marker data were available for only 46% and 36% of patients, respectively, and these variables were not included in the multivariate analysis to avoid a substantial reduction in sample size and loss of statistical power for other covariates.

## 4. Discussion

In this retrospective study, we evaluated the real-world effect of adjuvant chemotherapy in patients with resected SBA. Although the differences in DFS and OS between patients receiving adjuvant chemotherapy and those under observation were not statistically significant, the results indicated a trend toward improved survival outcomes in the selected high-risk subgroups. This finding adds to the limited but growing body of evidence supporting the potential benefits of adjuvant treatment for this rare malignancy.

The median DFS was modestly longer in the adjuvant chemotherapy group compared with the observation group, although the difference was not statistically significant. However, patients receiving adjuvant chemotherapy demonstrated higher rates of lymph node positivity and positive surgical margins compared with those managed with observation alone. We acknowledge that these imbalances could have stemmed from important confounding factors that may have attenuated the observed treatment effect. To address this, we performed a multivariate Cox regression analysis adjusting for potential confounders, including pathological N stage and margin status. Similarly, the adjusted HR of 0.81 from the multivariate model did not demonstrate a significant benefit. In particular, the lymph node-positive subgroup showed a tendency toward improved DFS with adjuvant chemotherapy (median OS, 37.5 vs. 23.6 months). However, a subgroup analysis according to margin status could not be performed owing to the absence of margin-positive cases in the observation arm. These factors may have partially masked the potential benefits of adjuvant treatment in this cohort. This finding is consistent with previous reports [11,12,14,16]. Notably, the DFS outcomes in our study were lower than those reported in previous studies. These discrepancies may be because of the higher-risk profile of our cohort (80% were patients with T4 tumors and 11% were those with margin-positive resections), compared with those in earlier studies [11,12,14]. The recurrence rate in our study (59%) was also substantially higher than that reported previously (26–38%), further underscoring the aggressive nature of the disease in our population.

Additionally, there was heterogeneity in the adjuvant treatment regimens which included both single-agent and doublet fluoropyrimidine-based chemotherapy, as well as a small proportion of patients received concurrent chemoradiotherapy. Only 45% of the patients in our cohort received a doublet chemotherapy regimen, compared with 54–69% in earlier studies [11,12,14]. This treatment heterogeneity may have contributed to differences in efficacy. However, owing to the small sample size, subgroup analyses based on the regimens were not feasible. In contrast, another retrospective study reported a significant DFS benefit (34 vs. 16 months; *p* = 0.001), with 78% of patients receiving combination chemotherapy [10]. However, their study lacked detailed reporting of margin status and baseline imbalances, limiting comparability. Taken together, although our study did not demonstrate a statistically significant DFS benefit, the consistent direction of the effect estimates supports the consideration of adjuvant chemotherapy in appropriate patients. Patient risk stratification and treatment regimens are important factors underlying the variability in results across studies.

In terms of OS, the median OS was longer in the adjuvant chemotherapy group compared with the observation group, although the difference was not statistically significant. Similar trends were observed for the 1-, 2-, and 3-year OS rates. These findings are consistent with those of previous studies that found no significant OS benefits [12,14,15,16]. Conversely, some reports have shown positive OS results from adjuvant chemotherapy only in patients with stage III disease [7,9]. In our cohort, the subgroup analysis suggested numerically improved OS in patients with T4 or node-positive tumors, although this was limited by the small sample size.

Notably, OS can be confounded by the subsequent lines of treatment for early stage cancers. In our cohort, 66% of the patients received palliative chemotherapy after recurrence, with a higher proportion in the adjuvant group (78.9%) than in the observation group (50.0%). This difference may have contributed to the observed OS. These data, which are often lacking in earlier studies, underscore the importance of considering post-recurrence management when interpreting OS outcomes. One patient in this cohort underwent a liver metastasectomy and achieved an excellent outcome. Although the definitive role of local treatment, including metastasectomy, in SBA remains limited, some reports have demonstrated promising outcomes [17,18,19]. Hence, a multidisciplinary team should be involved in the management of selected patients with limited metastatic disease to consider the potential role of further local treatments in improving survival outcomes on a case-by-case basis.

Subgroup analyses revealed that the potential benefits of adjuvant chemotherapy were more pronounced in patients aged < 65 years and in those with non-underweight BMI, T4 tumors, and node-positive disease. Although these differences were not statistically significant, they highlight specific high-risk populations that may derive greater benefits from systemic therapy. Given the small sample size and retrospective design, the study was underpowered to detect modest survival differences, and the observed nonsignificant trends may reflect a type II error. These findings should be interpreted with caution and considered hypothesis-generating. Previous findings have suggested benefits of adjuvant chemotherapy in high-risk stage III SBA [8]. These findings align with those of previous studies on gastrointestinal malignancies, where higher stage and nodal involvement correlated with increased recurrence risk and greater benefit from chemotherapy [20,21,22].

Regarding recurrence patterns, distant metastasis was the predominant mode of failure, most commonly involving the peritoneum, followed by the distant lymph nodes and liver, which is consistent with previous reports [4,14]. These findings suggest that systemic disease control remains a major therapeutic challenge in SBA; efforts to optimize systemic therapy are warranted.

The analysis also identified positive surgical margins as a significant predictor of inferior DFS, and pathological T4 stage as a poor prognostic factor for OS. These results are consistent with those of previous studies [3,13,23,24], and reinforce the importance of achieving complete resection and recognizing high-risk pathological features. Other factors reported to be associated with poor prognosis in other studies included duodenal location, lymph node involvement, and LVI [3,13,25,26]. Molecular and biomarker profiling (e.g., mismatch repair status, *KRAS* mutation, and HER2 overexpression) should be further explored in future studies; these data were unavailable for most patients in the present study because such testing was not routinely performed at our center during the study period. Although elevated levels of tumor markers (CEA and CA19-9) were associated with worse outcomes in univariate analyses, they were excluded from multivariate modeling because of missing data in over half of the cohort. Given the limited sample size, baseline imbalances, and retrospective design, these findings should be interpreted with caution as exploratory rather than as confirmatory results. Future prospective studies should incorporate these markers systematically as they may serve as valuable prognostic or predictive tools.

Despite inherent limitations, including its retrospective design, which might introduce selection bias, single-center setting, and small sample size, this study offers valuable real-world insights into the role of adjuvant chemotherapy in resected SBA. Given the rarity of the disease, RCTs remain difficult to conduct; thus, data from real-world settings are essential for clinical decision-making. Heterogeneity in chemotherapy regimens limits conclusions regarding the optimal treatment, and data on adverse events, toxicity, and quality of life were not collected. These are critical in an adjuvant setting, where the balance between benefits and harm must be carefully weighed. Another limitation is the relatively short overall median follow-up, which could underestimate long-term recurrence and survival outcomes, and the longer median follow-up in the adjuvant chemotherapy group, likely due to more recent treatment in the observation group. Differences in the follow-up duration between the treatment groups may have introduced potential immortal time bias. However, because of the small sample size, landmark analysis was not feasible, and hence this bias could not be fully eliminated. This difference may affect recurrence detection and survival comparisons. Additionally, information regarding whether resections were performed urgently or electively was unavailable; this may have influenced postoperative outcomes and the likelihood of receiving adjuvant therapy.

Larger, i.e., multi-institutional and multi-national, registry-based studies are warranted to better inform clinical decision-making regarding SBA. Collaborative efforts across countries would enable the assembly of sufficiently large and diverse patient cohorts, thereby enhancing the statistical power and generalizability of findings. Prospective cohort studies with standardized treatment protocols, longer follow-up periods, and systematic collection of toxicity and quality-of-life data would strengthen the evidence base. Furthermore, the integration of molecular and genomic profiling enables biomarker-driven treatment selection and risk stratification, thereby allowing for more personalized therapies. Investigations of the most effective adjuvant regimens, particularly in high-risk subgroups, are also required to optimize patient outcomes.

## 5. Conclusions

In this real-world single-center study of resected SBA, adjuvant chemotherapy showed a trend toward improved DFS and OS, compared with observation alone. However, trends favoring adjuvant therapy were observed in patients with T4 tumors, nodal involvement, younger age, and better nutritional status. Positive surgical margins and pathological T4 stage were associated with worse DFS and OS, respectively. Given the small sample size, baseline imbalances, and relatively short follow-up, these findings should be interpreted with caution. The decision to offer adjuvant chemotherapy should be individualized, balancing potential benefits and risks. Future larger multi-institutional and multi-national studies are warranted to clarify the role of adjuvant chemotherapy and to focus on biomarker discovery, regimen optimization, and patient-centered outcomes to improve the care for this rare but challenging disease.

## Figures and Tables

**Table 1:** Baseline characteristics.

	Adjuvant Chemotherapy (*n* = 29)	Observation (*n* = 23)	Total (*n* = 52)	*p*-Value
Sex, *n* (%)				1.0
Female	14 (48.3)	12 (52.2)	26 (50.0)	
Male	15 (51.7)	11 (47.8)	26 (50.0)	
Age, years (SD)	58.9 (12.7)	62.3 (13.2)	60.4 (12.9)	0.353
Age ≥ 65 years, *n* (%)	10 (34.5)	10 (43.5)	20 (38.5)	0.707
BMI, *n* (%)				0.819
<18.5 kg/m^2^	9 (31.0)	9 (39.1)	18 (34.6)	
18.5–22.9 kg/m^2^	12 (41.4)	8 (34.8)	20 (38.5)	
≥23.0 kg/m^2^	8 (27.6)	6 (26.1)	14 (26.9)	
Location, *n* (%)				0.889
Duodenum	21 (72.4)	18 (78.3)	39 (75.0)	
D1	1 (3.4)	0 (0)	1 (1.9)	
D2	17 (58.6)	16 (69.6)	33 (63.5)	
D3	3 (10.4)	2 (8.7)	5 (9.6)	
Jejunum	6 (20.7)	3 (13.0)	9 (17.3)	
Ileum	2 (6.9)	2 (8.7)	4 (7.7)	
Pathological T stage, *n* (%)				0.831
T3	5 (17.2)	5 (21.7)	10 (19.2)	
T4	24 (82.8)	18 (78.3)	42 (80.8)	
Pathological N stage, *n* (%)				0.005
N0	9 (31.0)	17 (73.9)	26 (50.0)	
N1	11 (38.0)	5 (21.7)	16 (30.8)	
N2	9 (31.0)	1 (4.3)	10 (19.2)	
Tumor differentiation, *n* (%)				0.367
Well	11 (37.9)	11 (47.8)	22 (42.3)	
Moderately	13 (44.8)	6 (26.1)	19 (36.5)	
Poorly	5 (17.2)	6 (26.1)	11 (21.2)	
LVI, *n* (%)	16 (55.2)	10 (43.5)	26 (50.0)	0.577
PNI, *n* (%)	5 (17.2)	5 (21.7)	10 (19.2)	0.734
Resection type, *n* (%)				0.391
Whipple operation	17 (58.6)	17 (73.9)	34 (65.4)	
Segmental resection	12 (41.4)	6 (26.1)	18 (34.6)	
Margin status, *n* (%)				0.028
Positive	6 (20.7)	0 (0)	6 (11.5)	
Negative	23 (79.3)	23 (100.0)	46 (88.5)	
CEA level, *n* (%)	(*n* = 15)	(*n* = 9)	(*n* = 24)	0.130
<5 ng/mL	14 (93.3)	6 (66.7)	20 (83.3)	
≥5 ng/mL	1 (6.7)	3 (33.3)	4 (16.7)	
CA19-9 level, *n* (%)	(*n* = 10)	(*n* = 9)	(*n* = 19)	0.350
<37 U/mL	8 (80.0)	5 (55.6)	13 (68.4)	
≥37 U/mL	2 (20.0)	4 (44.4)	6 (31.6)	

SD, standard deviation; BMI, body mass index; LVI, lymphovascular invasion; PNI, perineural invasion; CEA, carcinoembryonic antigen; CA19-9, carbohydrate antigen 19-9.

**Table 2:** Treatment information.

Treatment Information	*N* (%)
Regimen, *n* (%)	
Chemotherapy	26 (89.7)
FOLFOX	7 (24.1)
CAPOX	5 (17.2)
5-FU	11 (37.9)
Capecitabine	2 (6.9)
TS-one	1 (3.4)
Concurrent chemoradiotherapy	3 (10.3)
Cisplatin plus 5-FU + RT	1 (3.4)
5-FU + RT	1 (3.4)
Capecitabine + RT	1 (3.4)
Discontinuation, *n* (%)	
Complete treatment	20 (69.0)
Tumor recurrence	3 (10.3)
Patient preference	3 (10.3)
Adverse events	3 (10.3)

FOLFOX, folinic acid, fluorouracil, and oxaliplatin; CAPOX, capecitabine and oxaliplatin; 5-FU, 5-fluorouracil; TS-one, tegafur, gimeracil, and oteracil potassium; RT, radiotherapy

**Table 3:** Recurrence pattern and subsequent treatment.

	Adjuvant Chemotherapy (*n* = 29)	Observation (*n* = 23)	Total (*n* = 52)	*p*-Value
Recurrence, *n* (%)	19 (65.5)	12 (52.2)	31 (59.6)	0.491
Site of recurrence, *n* (%)				0.611
Local recurrence	1 (5.3)	2 (16.7)	3 (9.7)	
Distant recurrence	17 (89.4)	9 (75.0)	26 (83.9)	
Both local and distant recurrence	1 (5.3)	1 (8.3)	2 (6.4)	
Site of recurrence, *n* (%)				
Surgical bed/anastomosis	2 (10.5)	3 (25.0)	5 (16.1)	0.350
Peritoneum	8 (42.1)	6 (50.0)	14 (45.2)	0.952
Lymph node	5 (26.3)	5 (41.7)	10 (32.3)	0.447
Lung	2 (10.5)	4 (33.3)	6 (19.4)	0.174
Liver	5 (26.3)	3 (25.0)	8 (25.8)	1.000
Abdominal wall	1 (5.3)	1 (8.3)	2 (6.5)	1.000
Bone	0 (0)	1 (8.3)	1 (3.2)	0.387
Pleura	0 (0)	1 (8.3)	1 (3.2)	0.387
Second primary colon/colonic metastasis	1 (5.3)	1 (8.3)	2 (6.5)	1.000
Subsequent treatment, *n* (%)				
Palliative chemotherapy	15 (78.9)	6 (50.0)	21 (67.7)	0.127
Metastasectomy	1 (3.6)	0 (0)	1 (2.0)	1.000

**Table 4:** Prognostic factors.

Factor	DFS				OS			
	Univariate		Multivariate		Univariate		Multivariate	
	HR (95% CI)	*p*-Value	HR (95% CI)	*p*-Value	HR (95% CI)	*p*-Value	HR (95% CI)	*p*-Value
Age ≥65 years	1.3 (0.67–2.52)	0.44	-	-	1.7 (0.86–3.37)	0.127	2.04 (0.94–4.45)	0.072
Male vs. female	0.83 (0.43–1.59)	0.574	-	-	0.72 (0.37–1.42)	0.343	-	-
BMI (kg/m^2^)								
<18.5	1.66 (0.77–3.57)	0.192	1.67 (0.73–3.79)	0.221	1.14 (0.52–2.52)	0.738	-	-
18.5–22.9	Ref	-	Ref	-	Ref	-	-	-
≥23	0.96 (0.41–2.23)	0.924	0.81 (0.34–1.93)	0.63	0.89 (0.38–2.07)	0.791	-	-
Poor differentiation vs. well/moderate differentiation	1.27 (0.6–2.7)	0.535	-	-	1.1 (0.5–2.43)	0.814	-	-
T4 vs. T3	2.24 (0.86–5.83)	0.1	1.93 (0.7–5.36)	0.206	2.67 (0.93–7.65)	0.068	3.33 (1.04–10.6)	0.042
Positive lymph node	0.96 (0.5–1.85)	0.906	-	-	0.92 (0.47–1.80)	0.800	-	-
Positive LVI	1.67 (0.87–3.24)	0.126	1.66 (0.82–3.36)	0.156	1.56 (0.79–3.06)	0.200	1.41 (0.68–2.92)	0.350
CEA ≥ 5 ng/mL	13.63 (2.19–84.87)	0.005	-	-	26.65 (2.64–268.6)	0.005	-	-
CA19-9 ≥ 37 U/mL	4.58 (1.29–16.28)	0.019	-	-	2.30 (0.66–7.97)	0.190	-	-
Positive marginal resection	3.06 (1.12–8.34)	0.029	3.27 (1.11–9.62)	0.031	2.47 (0.93–6.54)	0.070	2.13 (0.70–6.50)	0.182
Location: duodenum vs. others	1.62 (0.74–3.56)	0.231	-	-	1.84 (0.8–4.23)	0.150	2.03 (0.83–4.93)	0.119
Adjuvant chemotherapy	0.86 (0.45–1.64)	0.642	0.73 (0.35–1.53)	0.403	0.63 (0.32–1.23)	0.179	0.54 (0.25–1.17)	0.117

DFS, disease-free survival; OS, overall survival; HR, hazard ratio; CI, confidence interval; BMI, body mass index; LVI, lymphovascular invasion; CEA, carcinoembryonic antigen; CA19-9, carbohydrate antigen 19-9.

## Data Availability

The datasets used and/or analyzed in the current study are available from the corresponding author upon reasonable request.

## Supplementary Materials

The following supporting information can be downloaded at https://www.mdpi.com/article/10.3390/jcm14217513/s1: Table S1. Details of palliative treatments.

## Abbreviations

The following abbreviations are used in this manuscript:

SBA	small bowel adenocarcinoma
DFS	disease-free survival
OS	overall survival
BMI	body mass index
LVI	lymphovascular invasion
PNI	perineural invasion
CEA	carcinoembryonic antigen
CA 19-9	carbohydrate antigen 19-9
HR	hazard ratio
CI	confidence interval
RCTs	randomized controlled trials
