# Supplementary material for: Role of Adjuvant Chemotherapy in Resected Small Bowel Adenocarcinoma: An Exploratory Real-World Analysis of Survival Outcomes and Prognostic Factors
Source: J Clin Med. 2025 Oct 23;14(21):7513. doi: 10.3390/jcm14217513 (PMC12608280; doi:10.3390/jcm14217513)
Supplement: Supplementary file 1 [file jcm-14-07513-s001.zip › jcm-3914033-supplementary.pdf]

Supplementary Material

Table S1 Details of palliative treatments

	Adjuvant chemotherapy (n=15)	Observation alone (n=6)
**Palliative chemotherapy, n(%)**		
FOLFOX	1 (6.7)	1 (16.7)
FOLFIRI	7 (46.7)	0 (0)
CAPOX	3 (20.0)	0 (0)
Cisplatin plus 5-FU	0 (0)	3 (50.0)
Carboplatin plus 5-FU	0 (0)	1 (16.7)
Cisplatin plus gemcitabine	0 (0)	1 (16.7)
DCF	1 (6.7)	0 (0)
5-FU	2 (13.3)	0 (0)
Gemcitabine	1 (6.7)	0 (0)

FOLFOX, folinic acid, fluorouracil, and oxaliplatin; FOLFIRI, folinic acid, fluorouracil, and irinotecan; CAPOX, capecitabine and oxaliplatin; 5-FU, 5-fluorouracil; DCF, docetaxel, cisplatin, and 5-fluorouracil.
